# Case of Spasm of Muscles of Face and Cataract Due to Dental Irritation

**Published:** 1884-08

**Authors:** Henry Sewill


### SELECTIONS.

### Case of Spasm of the Muscles of the Face and Cataract Due to Dental Irritation.

BY HENRY SEWILL, M.R.C.S., L.D.S.

Read before the Harveian Society, of London, and published in the British Medical Journal.

The patient was a middle-aged lady, unmarried. She had suffered all her life from bad teeth and neuralgia. During late years the suffering had frequently been acute, and she had a constant succession of alveolar abscesses, with great swelling of the face, especially on the right side. She had long been under the care of Dr. W. Hoffmeister, of Cowes, who had frequently urged her to have her teeth attended to, but without avail. The patient could not overcome her dread of dental operations.

About two years and a half ago, during 1881, her right eye became painful at times, apparently from neuralgia. In January, 1882, contraction of the muscles of that side of the face took place, and has persisted ever since. In July of that year, drooping of the right eyelid came on suddenly, and she then discovered that she had lost the sight of her right eye. Dr. Hoffmeister, believing that her condition was due to dental disease, and having again urged her in vain to have her teeth examined, sent her to Dr. Ferrier. In July, 1883, she went to Dr. Ferrier. He reported the case as follows:

She was a very nervous middle-aged spinster, with facial spasm and apparent neuralgia on the right side of the face and head.

She said she could not open her right eye, but on examination this was found to be not exactly true, as with effort she could do so. The closure of the eye was found to be due to spasm of the orbicularis muscle, and was not ptosis. She would scarcely allow her head and face to be touched, but there was evidently great tenderness in the region of the right upper canine. The neighboring teeth were much encrusted with tartar, and the gums looked red and swollen. The right lens was almost opaque.

Believing that the cause of the neuralgia, the facial spasm, and the cataract was in the dental disease, Dr. Ferrier insisted that she should have the teeth set right or extracted. That the dental disease was the cause of trophic disorder, leading to cataract, Dr. Ferrier considered most probable, looking at all the circumstances of the case, and the fact that the symptoms were entirely unilateral.

The patient was at length induced to have her teeth examined, and came to me. I found in the upper jaw the stumps of some molars, and the bicuspids, cuspids, and incisors, except the left central and lateral incisor. In place of these was a vulcanite palate, with artificial teeth attached to their neighbors. This had not been removed for years, and, as well as the rest of the teeth, was covered with tartar. The upper teeth were all carious, and were the centres of inflammation of long standing. The right cuspid was broken down nearly to the gum. Considerable thickening and swelling existed upwards over the root, and pus could be pressed from the gums through small fistulous openings over the roots of all the teeth. The lower teeth, of which about ten remained in front, were hidden beneath an enormous mass of tartar, which extended fully half an inch over the floor of the mouth. The patient's confession that she had endured years of incessant pain and discomfort from her teeth, through dread of dental operations, was amply born out by examination. Nitrous oxide was administered on two occasions, and the upper teeth were extracted. The tartar was gradually removed from the lower teeth, and an artificial set of upper teeth was provided.

Within two days of the extraction of the teeth the facial spasm entirely disappeared. The patient's health rapidly improved, but no change took place in the damaged lens.

This is the first case of disease of the eyeball with which I have met. I see cases of neuralgia in the neighborhood of the eye very commonly, and cases of neuralgia of the eyeball less frequently, in which the cause is easily traceable to the teeth, the eye being perfectly healthy. The pain, in some cases, is attended with lacrymation; in a few with muscular twitchings of the face. The most frequent condition of the teeth giving rise to neuralgia and other symptoms is caries, with exposure and chronic imflammation of the nerve. Patients are often unconscious of the condition of the teeth, the pain being distant and the teeth not aching. Touching the exposed nerve, or directing a stream of cold water upon the tooth, or into the cavity, will usually bring on a paroxysm of the distant pain, and establish a diagnosis where doubt exists. Dental periostitis, exostosis, and also impacted lower wisdom teeth are less common causes of neuralgic pain.

From a paper lately read at the Odontological Society, by Mr. Henry Power, ([1]) I learned, with considerable surprise, it must be confessed, that Mr. Power and numerous first-rate ophthalmic authorities, British and foreign, hold the opinion that dental lesions are very frequently the cause of serious diseases of the eye.

Mr. Power lays it down as a maxim to be generally observed that in all cases of threatening glaucoma, especially when this is associated with ciliary neurosis, and obscure pains in the temples and maxillary orbital regions; in all cases of mydriasis, and probably of myotis, originating without apparent cause; in all cases of sudden paralysis of either of the orbital muscles, or of the loss of sensation, in the absence of cerebral symptoms; in all cases of phlyctenular disease of the conjunctiva; in all cases of ulcers of the cornea resisting ordinary treatment; in all cases of sudden failure of accomodation, especially in young children; and, finally, in all cases of exophthalmia, the condition of the teeth should at least be examined, and if faulty conditions present themselves, these should be at once rectified, and then one, at least of the possible causes of each of these diseases will be removed. To this list must be added cataract; for, although Mr. Power tells me that he has not seen such a one, he accepts the case I have narrated as probably due to dental irritation.

1 "On the Relations Between Dental Lesions and Diseases of the Eye." By Henry Power. Transactions of the Odontological Society, November, 1883.

Mr. Power gives many cases of his own and of other well known observers — Hermann Schmidt, Galezowski, and von Grafe—in support of his opinions, and these opinions were corroborated by other ophthalmic surgeons in the discussion at the Odontological Society. Mr. Power points out the various ways in which the diseased action about the teeth may reach the eye.

These are (1) by inhibition; (2) by trophic or vaso-motor influence (under this heading Mr. Power would place my case); (3) by extension of neuritis, by reflex irritation or sympathy.

I must express no opinion on these views, but I may repeat that I have never, with one exception, met with a case of eye disease in which there seemed reason to suspect diseased teeth as a cause, that is, I have never had such a case referred to me as a dental surgeon in hospital or private practice.

I trust that my case may be considered worthy of record and of discussion, and, in order to stimulate discussion, I have ventured to cite the recent apropos remarks of Mr. Henry Power. —*British Medical Journal.*

